# Efficacy of Transdermal Nitroglycerin Patch for the Arrest of Preterm Labor: A Retrospective Study

**DOI:** 10.7759/cureus.59982

**Published:** 2024-05-09

**Authors:** Vidya Gaikwad, Suhas Gaikwad, Pragya Tiwari

**Affiliations:** 1 Obstetrics and Gynecology, Dr. D. Y. Patil Medical College, Hospital & Research Centre, Dr. D. Y. Patil Vidyapeeth (Deemed to be University) Pimpri, Pune, IND

**Keywords:** uterine contractions, effacement and dilation, cervix, nitroglycerine patch, preterm labor

## Abstract

Background

Transdermal nitroglycerin (NTG) is a potent smooth muscle relaxant acting as a tocolytic agent by acting on the uterine muscles. The transdermal patch allows for continuous and controlled release of NTG through the skin into the bloodstream. This method offers the advantage of sustained drug delivery over a prolonged period.

Objective of the study

The study aimed to evaluate the efficacy of NTG patches for the arrest of preterm labor.

Materials and methods

This retrospective study comprised 100 patients admitted to our tertiary care center, ranging from 27 to 35 weeks of gestation, exhibiting preterm labor, uterine contractions, effacement, and dilatation of the cervix, without comorbidities and complications.

Results

In this study, it was observed that the incidence of preterm labor was higher among women aged 21-25 years. Pregnancy duration was extended by an average of approximately 28.63 days in our study cohort, with 90% of patients experiencing a prolongation of pregnancy to 48 hours after the application of a transdermal NTG patch. Parity distribution showed 50% of patients having a parity of G2-G4 and 30% being primigravida. However, 40% of the participants reported experiencing side effects, including headaches (15%) and local reactions (25%), while 60% did not experience any adverse effects.

Conclusion

In this study we found that the application of transdermal NTG patches led to a mean prolongation of pregnancy by 28.63 days, allowing time for the administration of steroids and fetal maturation. The inhibition of preterm contractions was successful, with an efficacy rate of 92%. These findings suggest the potential effectiveness of transdermal NTG patches as a tocolytic agent in managing preterm labor. However, the occurrence of side effects highlights the importance of careful monitoring and management during treatment.

## Introduction

Preterm birth poses a significant medical and socioeconomic challenge globally, representing the leading cause of morbidity and mortality in neonates [1]. Although neonatal intensive care has advanced, the associated healthcare costs have escalated, emphasizing the dual nature of preterm birth as both a medical and socioeconomic issue [2]. Accounting for a substantial portion of perinatal mortality and morbidity, estimated at 75-80%, preterm birth underscores the imperative for obstetricians to accurately predict and effectively prevent premature labor. In India, the incidence of preterm birth ranges from 10% to 12%, mirroring the global prevalence of 11%, highlighting the widespread impact of this issue on maternal and child health [3,4].

Research indicates that for pregnancies between 28 and 34 weeks, delaying delivery by a day results in a 3% increase in survival rates for the neonate. This underscores the importance of delaying preterm delivery through tocolysis, allowing sufficient time for the administration of the full course of antepartum steroids. In addition, delaying delivery facilitates in-utero transfer to a tertiary care center, where neonatal care is optimal, further enhancing the chances of favorable outcomes for both the mother and the newborn [5].

The outcome and complications in preterm babies are closely linked to gestational age, with earlier gestational ages associated with higher risks of adverse outcomes. Various factors contribute to preterm labor, including infections, such as local vaginal infections, periodontitis, and urinary tract infections, along with other factors, like anemia, low socioeconomic status, teenage pregnancy, advanced maternal age, low BMI, and substance abuse, such as smoking and alcohol consumption. Anatomical abnormalities of the uterus and cervix, polyhydramnios, multiple pregnancies (twins), and placental complications are also known to contribute to preterm labor in some cases [6]. Identifying and addressing these risk factors are crucial in the prevention and management of preterm labor to improve outcomes for both the mother and baby.

Predictors of preterm labor encompass a range of factors, including multiple pregnancies, a history of prior preterm delivery, genital infections, anemia, and cervical length, along with various signs and symptoms [7]. Symptoms indicative of preterm labor may include lower back pain, abdominal pain, painful uterine contractions or tightening, and the passage of blood-stained vaginal discharge (known as "show"), as well as a sensation of pelvic or vaginal pressure. Clinical signs that may lead to the diagnosis of preterm labor include palpable uterine contractions occurring more than four times in 20 minutes, engagement of the presenting part, cervical dilation of more than 1 cm, cervical effacement, the presence of show, and bulging membranes. Early recognition and management of these signs and symptoms are crucial in preventing preterm birth and reducing associated complications for both the mother and baby [8].

Effective preterm labor prevention requires a multifactorial approach that includes public education programs, lifestyle modifications, improved access to healthcare, and effective antenatal care (ANC). Early diagnosis and management are essential components of this approach. Tocolysis, which involves the use of medications to inhibit uterine contractions, plays a crucial role in managing preterm labor by allowing time for the administration of steroids to enhance fetal lung maturity, thus improving fetal outcomes [9]. While several tocolytic drugs are available, none has demonstrated a clear therapeutic advantage over others, emphasizing the need for individualized treatment strategies based on patient-specific factors and clinical judgment. This comprehensive approach aims to reduce the cases of preterm birth and mitigate its associated complications, ultimately improving maternal and neonatal health outcomes [10].

Nitroglycerin (NTG), a nitric oxide donor, is a potent smooth muscle relaxant acting as a tocolytic agent by acting on the uterine muscles. Administered via a transdermal patch, it has been observed to have minimal reported cardiovascular side effects. NTG functions as a vasodilator, aiding in maintaining normal smooth muscle tone in the uterus. It may offer a promising option for the management of preterm labor, contributing to improved maternal and neonatal outcomes [11].

NTG exhibits efficient absorption through various routes including buccal mucosa, intestines, and skin due to its lipid solubility. However, its metabolism in the liver, facilitated by a glutathione-dependent organic nitrate reductase, results in rapid metabolism of the active chemical. Transdermal administration circumvents this issue by ensuring sufficient plasma concentrations, thus optimizing its therapeutic effects [12]. With action commencing within 60 minutes of administration, NTG demonstrates a bioavailability ranging from 70% to 90%, indicating its potential as an effective tocolytic agent for managing preterm labor.

NTG, while effective as a tocolytic agent, comes with specific contraindications, including acute circulatory failure, hypotensive conditions, glaucoma, raised intracranial pressure, obstructive hypertrophic cardiomyopathy, and known sensitivity to nitrate compounds. In addition, caution is warranted in patients with advanced labor, maternal cardiac diseases, hypertensive disorders, diabetes mellitus, intrauterine growth restriction (IUGR), intrauterine fetal demise, antepartum hemorrhage (APH), or abnormal fetal well-being tests.

Various other tocolytic agents, such as isoxsuprine hydrochloride, ritodrine, magnesium sulfate, nifedipine, and atosiban, have been utilized to suppress uterine contractions, each with its own set of indications and contraindications [13-17]. However, the efficacy of any tocolytic drug lies in its ability to successfully halt premature uterine contractions while minimizing maternal and fetal side effects. Thus, careful consideration of the patient's medical history and individual risk factors is essential in determining the most suitable tocolytic therapy for optimal maternal and fetal outcomes.

## Materials and methods

This retrospective study, conducted at Dr. D. Y. Patil Medical College, Hospital & Research Centre, a tertiary care center in Pune, India, between 2018 and 2019, aimed to evaluate the effectiveness and efficacy of NTG in managing preterm labor. A total of 100 patients meeting the inclusion and exclusion criteria, aged between 27 and 36 weeks of gestation, were selected for the study. Informed written consent was obtained from all participants. Patients clinically diagnosed with preterm labor were administered a 10 mg NTG patch applied on the anterior abdominal wall, and contractions were assessed after two hours. If contractions persisted, another 10 mg patch was applied, and the patient was reassessed in two hours. If contractions failed to disappear, then another tocolytic agent was used. However, if contractions subsided with the NTG patch, then a 5 mg NTG patch was applied 24 hours later and kept for 24 hours.

Monitoring

During the acute tocolysis with the 5 mg NTG patch, the patients underwent rigorous monitoring as per the following protocol: Vital signs, including pulse rate, blood pressure, respiratory rate, and fetal heart rate, were assessed every 30 minutes for the initial two hours, followed by monitoring every six hours thereafter. In addition, uterine contraction frequency and strength were evaluated every 30 minutes for the first two hours and then every six hours subsequently. This intensive monitoring regimen aimed to closely observe the patient's response to the tocolytic treatment and ensure prompt identification of any adverse reactions or changes in maternal or fetal condition. 

Following the acute phase of treatment with the NTG patch, the patients transitioned to oral medication with tab Nitrocontin 2.5 mg BD for seven days. Subsequently, the patients were discharged and followed up in the antenatal care (ANC) setting for further monitoring and management.

Inclusion criteria

The inclusion criteria for the study encompassed pregnant women encountering preterm labor pain within the gestational age range of 27 to 35 weeks. In addition, the presence of painful uterine contractions meeting specific frequency criteria was required: either experiencing four contractions in 20 minutes or eight contractions in 60 minutes, with each contraction lasting for a minimum of 30 seconds. Furthermore, cervical effacement exceeding 80% and cervical dilatation falling between greater than 1 cm to less than 4 cm, all while maintaining intact membranes. These criteria collectively aimed to ensure the enrollment of pregnant individuals experiencing the clinical manifestations indicative of preterm labor, thereby aligning with the objectives of the study.

Exclusion criteria

The exclusion criteria for the study involved several factors aimed at ensuring the safety and appropriateness of participants for the intervention. First, women in active labor, characterized by cervical dilation surpassing 4 cm, were excluded. In addition, patients with systemic diseases and obstetric complications, such as rupture of the membranes, eclampsia, antepartum hemorrhage, chorioamnionitis, severe pregnancy-induced hypertension, or oligohydramnios, were also excluded.

Furthermore, exclusion criteria encompassed cases involving fetal demise or fetal congenital malformations. Individuals exhibiting hypotension, indicated by systolic blood pressure below 90 mmHg, were excluded due to potential safety concerns related to the administration of NTG. Finally, patients with known sensitivity or contraindication to NTG were excluded to mitigate the risk of adverse reactions or complications associated with the intervention.

Outcome

The study was conducted between 2019 and 2020 following approval from the institutional ethical committee. Success criteria were defined as the cessation of uterine contractions, no progression of cervical changes, completion of the course of antenatal corticosteroids, and absence of recurrent uterine contractions within 24-48 hours following the cessation of the NTG patch.

## Results

The distribution of study subjects by age group revealed that 36% were in the age group of 26-30 years, followed by 30% in the age group of 21-25 years. A smaller proportion, comprising 11% of the study subjects, fell within the age group of 18-20 years, and 23% of the subjects were more than 31 years of age (Table 1).

**Table 1:** Distribution of the study subjects per age

Groups	Frequency	Percent (%)
18-20 years	11	11.0
21-25 years	30	30.0
26-30 years	36	36.0
>31 years	23	23.0
Total	100	100

In this study 52% of the study subjects were at a gestational age between 31 and 33 weeks, making this the most common gestational period among the participants. In addition, 27% of the subjects were in the gestational age group of 28-30 weeks, and 21% of the subjects were in the gestational age group of 34-37 weeks (Table 2).

**Table 2:** Distribution of the study subjects as per gestational age

Gestational age in weeks	Frequency	Percent (%)
28-30 weeks	27	27.0
31-33 weeks	52	52.0
34-37 weeks	21	21.0
Total	100	100.0

Table 3 illustrates the distribution of study subjects based on their parity status. It indicates that 50% of the study participants had a parity status of G2-G4, implying that they had previously experienced two to four pregnancies. In addition, 30% of the subjects were primigravida, so they were experiencing their first pregnancy. Lastly, 20% of the participants had a parity status greater than G4, indicating that they had more than four previous pregnancies.

**Table 3:** Distribution of the study subjects as per parity

Parity	Frequency	Percent (%)
Primigravida	30	30.0
G2-G4	50	50.0
>G4	20	20.0
Total	100	100.0

Furthermore, the efficacy of the NTG patch in abolishing premature uterine contractions while minimizing maternal and fetal side effects was also analyzed. The data indicate that the NTG patch demonstrated a remarkable efficacy rate of 92% in subsiding premature uterine contractions among the study participants (Table 4). This high efficacy suggests that the NTG patch is a promising intervention for managing preterm labor, highlighting its potential as a tocolytic agent with favorable outcomes for both maternal and fetal health.

**Table 4:** Distribution of cases by efficacy of the nitroglycerin (NTG) patch

Efficacy of the NTG patch	Frequency	Percent (%)
Yes	92	92%
No	8	8%
Total	100	100.0

After the application of NTG patches, the mean prolongation of pregnancy was found to be 28.63 ± 19.193 days (Table 5). This indicates that NTG patches effectively delay labor onset, providing additional time for interventions, such as administration of corticosteroids for fetal lung maturation and transfer to tertiary care centers for optimal neonatal care. Prolonging pregnancy by nearly a month can significantly improve neonatal outcomes by reducing the risk of prematurity-related complications and enhancing the overall health of the newborn.

**Table 5:** Number of days prolonged

Prolongation of pregnancy	Minimum	Maximum	Mean	SD
No. of days prolonged	7	66	28.63	19.193

Among the participants, 25% experienced a local reaction following the application of the NTG patches, which was managed with local calamine soothing lotion. In addition, 15% reported headaches, which were alleviated with simple analgesics (Tables 6, 7). Importantly, there were no instances of hypotension, tachycardia (heart rate >120 beats per minute), palpitations, or chest pain observed among the patients. These findings suggest that while some participants experienced mild side effects, they were manageable and did not pose significant risks to maternal health.

**Table 6:** Side effects of the nitroglycerin patch

Side effect	Frequency (%)
Yes	40 (40%)
No	60 (60%)
Total	100

**Table 7:** Side effects experienced

Side effects experienced	Frequency (%)
Headache	15 (37.5%)
Local reaction	25 (62.5%)
Total	40

## Discussion

**Figure 1:**
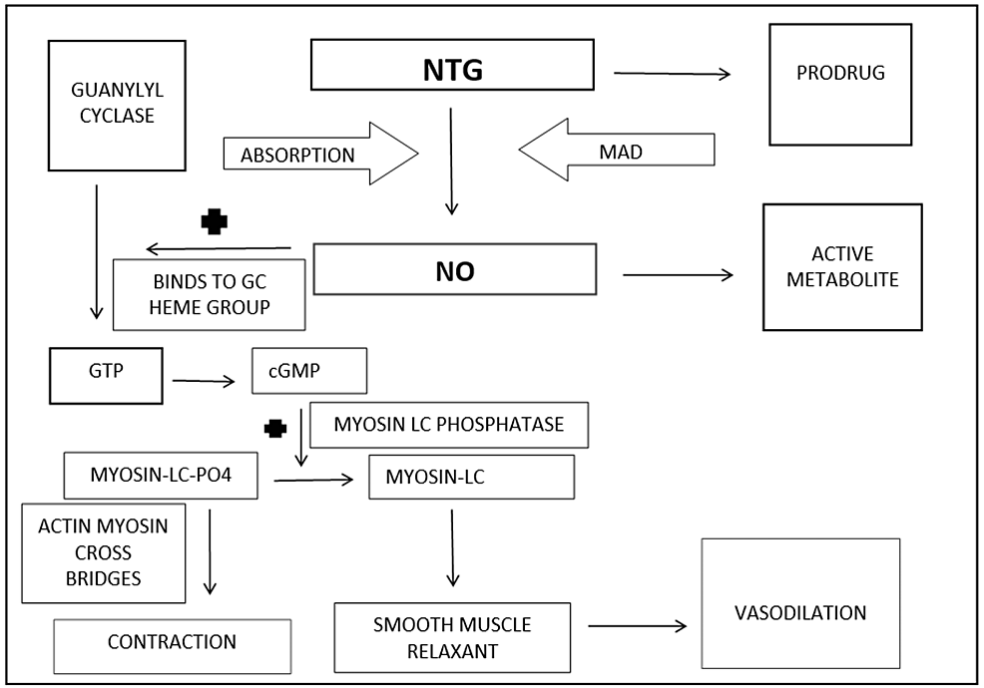
Mechanism of action of nitroglycerin (NTG) Modified from Toader et al. (2020) [18]

NTG is a promising option for the management of preterm labor being a potent smooth muscle relaxant, acting on the uterine muscles (Figure 1).

The objective of treating preterm labor is to extend the duration of pregnancy sufficiently to decrease the incidence of neonatal morbidity and mortality linked with prematurity. Premature newborns face elevated risks of morbidity and mortality due to their immaturity, leading to low birth weight and insufficient pulmonary surfactant production, resulting in conditions, such as respiratory distress syndrome. Studies have demonstrated a notable reduction in the occurrence of respiratory distress syndrome, intraventricular hemorrhage, and neonatal mortality following the administration of a complete course of corticosteroids. Therefore, prolonging pregnancy with an effective tocolytic agent is crucial to facilitate corticosteroid administration, suppress premature labor, or enable the transfer of patients to advanced care units when necessary. This study aimed to assess the efficacy of NTG patches as a tocolytic treatment for preterm labor, involving a total of 100 patients diagnosed with preterm labor.

The current study highlights a higher incidence of preterm labor among women aged 21-25 years, aligning with findings from the QUARISMA trial, which reported an increase in preterm births in the age group of 20-24 years. Notably, 52% of participants in our study were between 31 and 33 weeks of gestation, consistent with previous research by Afifa Waheed and Nabeela Shami in 2007 [19]. Another study by Kunjan Shah and colleagues demonstrated that in 76% of cases, the application of NTG patches extended pregnancy by more than seven days [20]. In the present study, pregnancy was prolonged for 48 hours in 90% of the patients and by an average of approximately 28.63 days. Previous research by Rowlands et al. in 1996 utilized a 50 mg NTG patch in a study to treat preterm cervical dilatation [21].

In 1994, Lees et al. conducted a study using a 10 mg glyceryl trinitrate patch to arrest preterm labor and prolong the gestation [22], which was similar to the approach adopted by Krishna et al. and in the current study. Parity distribution in this study revealed that 50% of the patients had parity G2-G4, with 30% being primigravida, aligning closely with findings from Aurooj et al.'s study in 2011, where 52.7% of the patients were of parity G2-G4 and 29.3% were primigravida [23]. Among the patients in this study, 25% experienced local reactions, such as redness, rash, and itching at the application site, managed with local calamine soothing lotion and site changes, while 15% reported headaches, which were alleviated with simple analgesics.

The transdermal nitroglycerin patch presents an attractive option for tocolysis due to its ease of administration, potential effectiveness, low cost, and minimal side effects. Its use as a tocolytic agent proved effective in preventing preterm labor, with the primary objective of delaying delivery by 48 hours to allow for the administration of steroids or arrangements for in-utero transfer if necessary. The transdermal route of administration offered better tolerance and acceptability among patients. Moreover, the nitroglycerin patch is cost-effective. This study underscores the effectiveness of the nitroglycerin patch as a tocolytic agent, demonstrating high efficacy in prolonging pregnancy with minimal maternal and fetal side effects.

Limitations

The major limitation of this study lies in the sample size, which can be expanded to strengthen the results and draw more definitive conclusions, particularly regarding the optimal dosage and regimen of tocolytics. Given that our study was conducted at a single hospital, studies involving a multicentre approach would provide more robust data. Furthermore, it is crucial to acknowledge that preterm labor is a multifactorial condition influenced by various unknown causative factors, which may impact the efficacy of tocolytics in preventing preterm labor. Therefore, future research should consider exploring these factors to enhance our understanding and management of preterm labor effectively.

## Conclusions

According to the findings of this study, the application of a transdermal NTG patch was effective in prolonging the pregnancy. There was a mean prolongation of pregnancy by 28.63 days. Furthermore, a substantial majority, comprising 90% of the subjects, experienced a prolongation of pregnancy to 48 hours post-application. This extended time frame provided a crucial window for the administration of steroids and facilitated fetal maturation. In addition, the inhibition of preterm contractions proved highly successful, demonstrating an impressive efficacy rate of 92%. These results underscore the effectiveness of transdermal NTG patches in managing preterm labor, offering promising prospects for improving pregnancy outcomes.
